# Determination of Mortar Strength in Historical Brick Masonry Using the Penetrometer Test and Double Punch Test

**DOI:** 10.3390/ma13122873

**Published:** 2020-06-26

**Authors:** Dawid Łątka, Piotr Matysek

**Affiliations:** Faculty of Civil Engineering, Cracow University of Technology, Warszawska 24, 31-155 Cracow, Poland; piotr.matysek@pk.edu.pl

**Keywords:** mortar compressive strength, penetrometer test, double punch test, minor destructive testing, brick masonry

## Abstract

This paper presents the results of the minor destructive testing of mortars in masonry structures of four buildings erected at the turn of the 19th and 20th centuries. The buildings were erected in the historical centre of Cracow. The objective of testing was to determine mortar compressive strength in masonry joints. The in situ tests were carried out with the use of a penetrometer RSM-15 with the standardised impact energy equalling 4.55 nm. Laboratory tests on mortar specimens taken from the structures were also performed. The double punch test method was used in the laboratory tests. On account of the specificity of the tested historical mortars, the typical procedures used in penetrometer and double punch tests were modified. For penetrometer tests, a new feature called “a surface disturbance zone” was introduced. Additionally, a procedure for determining a surface disturbance zone range was included. As confirmed in the paper, the consideration of the surface disturbance zone in the analysis of test results is crucial for the correct evaluation of mortar compressive strength. The thicknesses of bed joints in the tested historical masonry considerably exceeded the requirements included in the standard EN 1996-1-1. Thus, the thickness of the mortar specimens taken from historical masonry for the double punch tests clearly exceeded the thickness of specimens extracted from the typical structures erected nowadays. This article provides a method of considering a specimen thickness parameter in the analysis of double punch test results. The in situ test results with the use of penetrometer and double punch methods confirmed that the mortar strength in tested historical buildings ranged from 1.4 to 2.9 MPa. Mortar compressive strength values determined by both applied methods were similar.

## 1. Introduction

The basic structural elements of historical masonry buildings are walls and pillars subject to compression. The determination of the masonry mechanical parameters, such as compressive strength and Young’s modulus, is crucial in the analysis of these types of structures. Currently, various testing methods are applied in order to evaluate these parameters.

The best results are provided by in situ tests or by tests carried out on masonry specimens cut from the structure [1,2,3,4,5,6,7,8]. The masonry specimens’ dimensions and quantities should be sufficiently large in order to be representative of the structure [1,2,3,4]. Due to conservation reasons, satisfying this condition in every case is not possible because cutting out masonry specimens is connected with damaging historical structures. Furthermore, the process of cutting specimens out from walls erected on weak lime mortars may result in the splitting of specimens, making them useless in strength tests. Therefore, other methods of evaluating masonry compressive strength are applied as well.

A popular method of in situ masonry testing is a structural investigation with the use of flat-jacks. They are used to estimate the level of compressive stress in the structure and Young’s modulus of the masonry [5,6,7,8]. Nevertheless, the possibility of determining the masonry compressive strength is limited due to the damage to the fragment of the wall between the flat-jacks that usually appears during the test.

Brick masonry compressive strength is frequently estimated from formulas based on the strength of bricks and mortar [9,10]. The compressive strength of bricks may be obtained from laboratory tests conducted on bricks extracted from the structures. Such tests are usually performed on whole or half bricks. In order to minimise damage to historical structures, tests are also conducted on smaller brick specimens [11,12,13]. It is far more difficult to determine mortar strength in the masonry joints. On account of the dimensions of bed joints, it is not possible to cut out mortar specimens of dimensions required by EN 1015-11 [14], and due to this fact, it is not possible to carry out standard destructive tests on 4 × 4 × 16 cm^3^ mortar specimens. For this reason, minor destructive methods are used for the evaluation of mortar strength in historical brick walls. These methods are based on tests carried out on small specimens cut out from masonry bed joints or by in situ tests [15,16,17,18,19,20,21,22,23,24,25,26,27,28,29,30,31,32,33]. Most in situ methods are based on the measurement of the depth of steel needle penetration or the amount of energy required to drill a small cavity in the mortar joints. In situ and laboratory tests of mortars are in the process of being developed and tested in order to determine factors affecting the measurement results. Most frequently, the results of minor destructive testing of mortars are conducted on samples made of contemporary materials or materials whose properties are similar to those used in the historical mortars. There are significantly fewer tests performed on original, historical structures.

This article presents the compressive strength tests of mortars in the bed joints of masonry in historical buildings erected at the end of the 19th century and at the beginning of the 20th century in the centre of the royal city of Cracow. The mortar strength in the bed joints of masonry was defined with the use of a penetrometer and double punch tests. The penetrometer tests (PT) were carried out by means of an RSM-15 version 1.0 penetrometer [34]. The double punch tests (DPT) were performed in accordance with DIN 18555-9 [35]. A characteristic of the historical masonry was the considerable thickness of bed joints, the presence of large size grains in the mortar (diameters significantly exceeding 2 mm) and degradation of the mortar material in the areas near the external surface of the joints. Due to these factors, the authors of this article proposed modifications to the methods for determining the mortar compressive strength based on the penetrometer tests (PT) and double punch tests (DPT).

## 2. Experimental Research of Historical Mortars

### 2.1. Scope of Research

**Figure 1:**
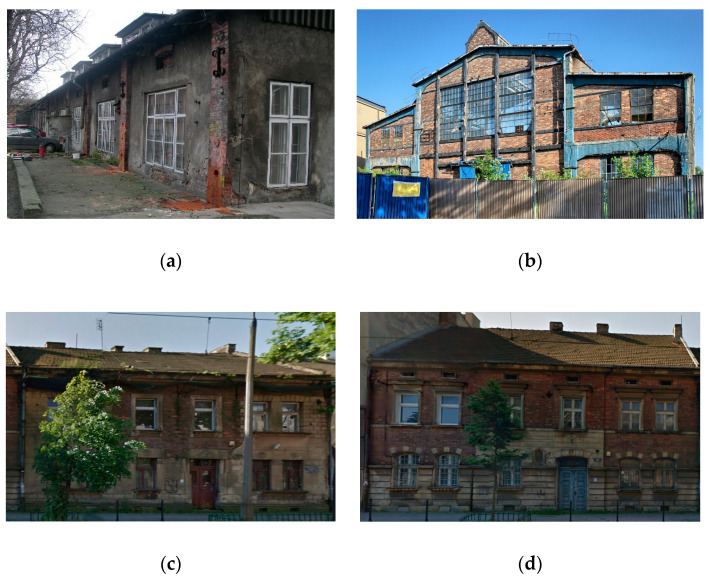
The facade-side view of the buildings: (**a**) warehouse building-B1; (**b**) manufacturing hall-B2; (**c**) tenement house-B3; (**d**) tenement house-B4.

The objects of testing were the mortars in the bed joints of four buildings erected at the turn of the 19th and 20th centuries in the historical centre of Cracow. The buildings are presented in Figure 1. The mortars were tested as part of projects aimed at identifying the construction materials used in the historical structures. The buildings are marked B1, B2, B3 and B4. The specification of the buildings is presented in Table 1.

The buildings marked B1 and B2 were used in the past as a warehouse and a factory, respectively. Buildings B3 and B4 were typical residential buildings, used for more than 100 years. In buildings B1, B3 and B4, the main construction elements were walls and pillars erected from solid clay bricks. In building B2, the main load-bearing structure was monolithic reinforced concrete structure, while the brick walls were the filling between the RC beams and columns.

The facades of buildings B2, B3 and B4 did not have any plastering. Buildings B1 and B2 had architectural elements like bossage and decorative moulding on the facade. On the inside, lime plastering was found on the masonry walls of buildings B1, B2, B3 and B4 (except for the basements in buildings B3 and B4).

In situ penetrometer tests were performed in all the buildings on the mortars in the masonry bed joints. At the same time, mortar specimens for the DPT tests were taken from bed joints. 

### 2.2. In Situ Tests Using Penetrometer (PT)

**Figure 2:**
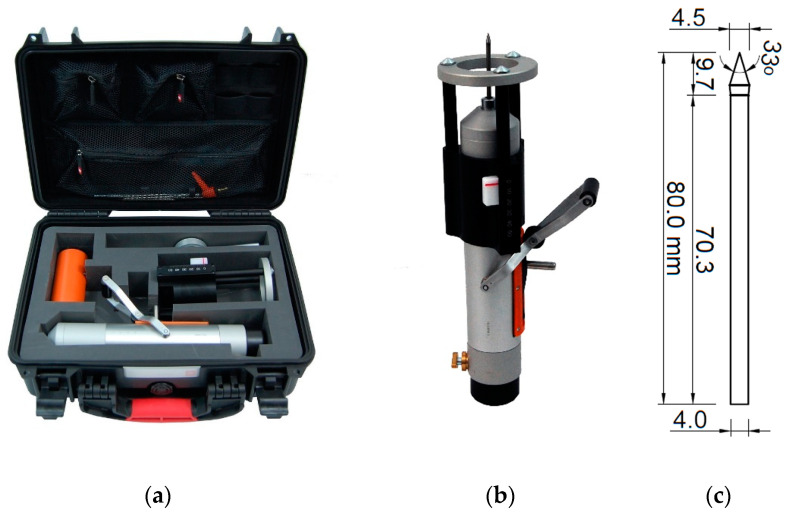
Penetrometer RSM-15 [34]: (**a**) complete testing set; (**b**) measurement device; (**c**) steel needle geometry.

In situ testing of the mortars in the bed joints of the masonry walls was carried out with the use of the impact penetrometer RSM-15 with the impact energy of 4.55 Nm (see Figure 2).

Testing by penetrometer consisted of measuring the penetration depth of the steel needle in the masonry joint as a result of the subsequent impacts of a mass actuated by a spring. The penetration depth measurement was carried out using the external measuring body (see Figure 2b), which provided the needle displacement. The penetration depth was measured in millimetres. 

**Figure 3:**
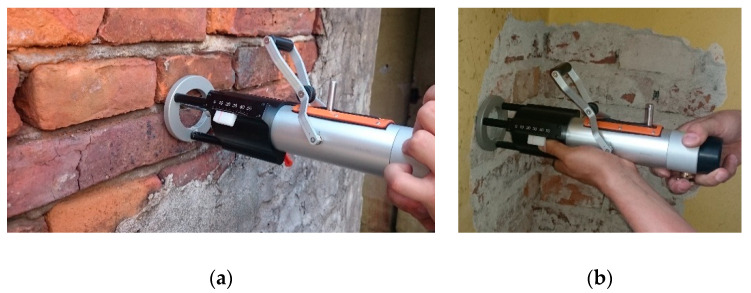
Penetrometer tests (PT)—the examples of test stands: (**a**) testing the mortar in the brick wall from the outside (non-plastered wall fragment); (**b**) testing the mortar in the masonry wall from the inside (after plaster removal).

The penetrometer tests in the historical buildings were performed on the surfaces of the walls. In each case, the testing area was precisely cleaned and levelled before performing the tests. For non-plastered wall surfaces (see Figure 3a), possible repointing materials and loose mortar fragments were removed. If the tests were to be conducted on the masonry wall from the inside, as shown in Figure 3b, lime plaster and levelling joints were removed through brushing before testing. After preparation of the testing area, the penetrometer was positioned perpendicular to the face of the masonry wall. The steel needle of the penetrometer was placed in the middle of the bed joint. According to the penetrometer manufacturer’s manual [34], the indications of the needle depth should be registered after every five subsequent impacts. In the authors’ tests, on account of the specificity of historical mortars, the indications of the needle depth were read out and noted after each impact.

**Figure 4:**
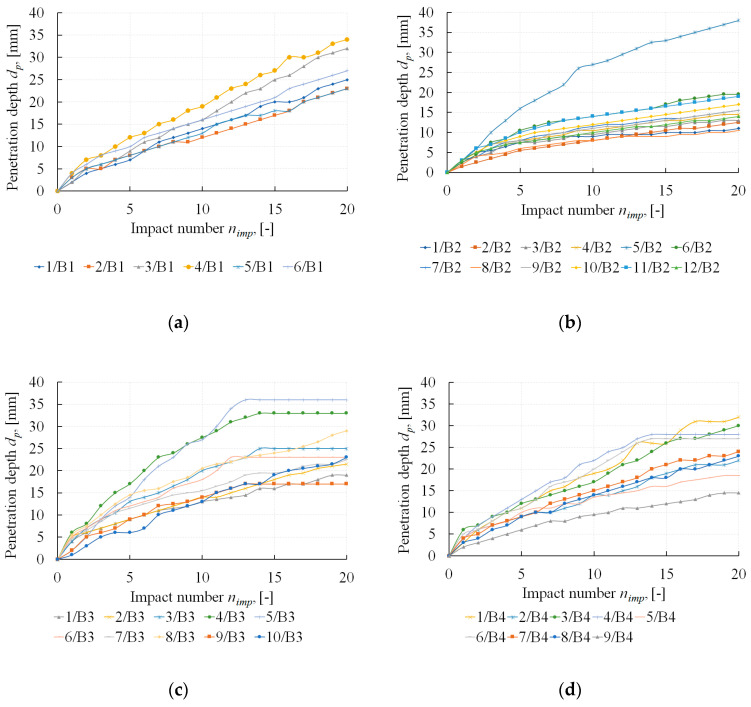
The results of penetrometer tests (PT)—the number of impacts vs. steel needle penetration depth: (**a**) tests in building B1; (**b**) tests in building B2; (**c**) tests in building B3; (**d**) tests in building B4.

The registered results of penetration depth are presented in Figure 4. While analysing the obtained functions of relations between the number of impacts (*n_imp_*) and the needle penetration depth (*d_p_*) in detail, it was noted that some curves clearly differed from the others. This resulted from two phenomena which occurred during the test. The first phenomenon was caused by the needle hitting a large size aggregate grain. In this case, the needle was blocked, and despite subsequent impacts, the needle did not penetrate into the joint. The result is a horizontal line, as shown in example curves 4/B3 and 5/B3 in Figure 4c. This phenomenon also occurred during testing low thickness joints or when bricks used for erecting a structure were extremely uneven on the bed side. 

The other phenomenon took place when the penetrometer needle was immersed into the mortar of a loose structure. The increase in the penetration depth for such a testing point was significantly higher than in the case of the testing points of joints that were filled properly. This can be seen in 5/B2 in Figure 4b. Due to the fact that both phenomena distorted the test results to a considerable degree, it was decided that curves affected by such phenomena would be omitted.

Finally, the following number of penetrometer tests were taken for further analysis: 6 tests for building B1, 10 tests for building B2, 8 tests for building B3 and 7 tests for building B4.

### 2.3. The Double Punch Tests (DPT)

**Figure 5:**
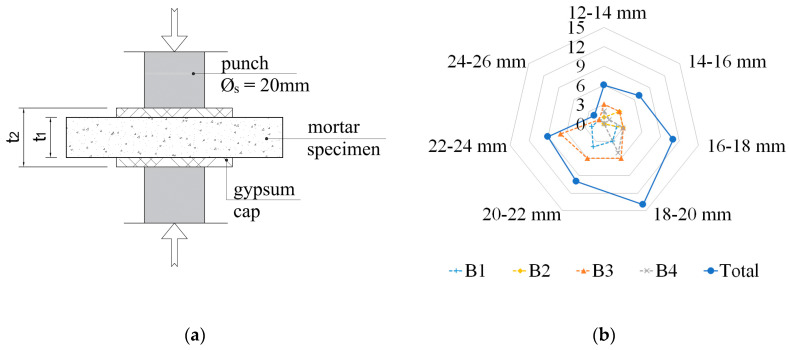
DPT tests: (**a**) the specimen loading scheme; (**b**) the specimen population structure depending on thickness *t*_1_.

The DPT tests were introduced for the first time by Henzel and Karl [16] in order to define the mechanical parameters of the specimens of mortars taken from a structure. This paper applied the test methodology, according to DIN 18555-9 [35]. The height of mortar specimens used in the DPT tests was equal to the bed joint thickness; whereas, the dimensions of specimens in the horizontal direction, in conformity with DIN 18555-9 [35], were approximately 50 mm. The mortar specimens were loaded by steel punches with a diameter of 20 mm. The specimen loading scheme is presented in Figure 5a. The mortar strength obtained in the DPT tests (*f_DPT_* expressed in MPa) was calculated as the ratio of maximum force to the area of the steel punch cross-section. A very similar DPT method was proposed in UIC-code [36], in which the diameter of the steel punch was recommended as 25 mm.

**Figure 6:**
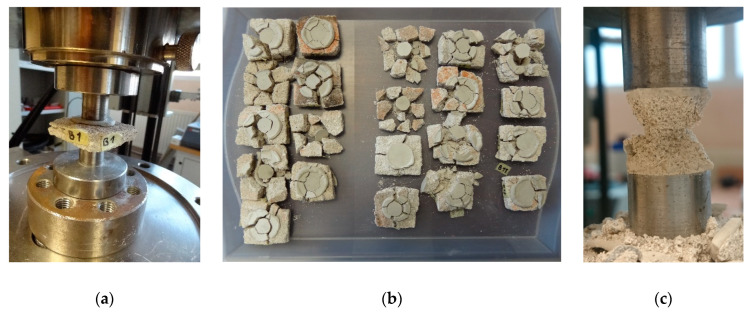
DPT test: (**a**) mortar specimen during loading; (**b**) the failure mode of specimens taken from building B3 and B4—top view; (**c**) a typical failure mode of the mortar specimen between steel punches.

The mortar specimens intended for the DPT tests were collected from the same building walls where PT tests were conducted. The specimens were collected by means of core drilling with the centrally positioned bed joint or through splitting the mortar from the fragments of walls intended for demolition. The joints were split from bricks by means of a steel chisel. The obtained irregular fragments of the bed joints were used to cut out square specimens with sides equalling approximately 50 mm. Then, on the lower and upper surface of the specimens, in the area of applying load, gypsum caps were made. After preparation of gypsum caps, the mortar specimens were centrally located in the testing setup and compressed up to failure. The mortar specimens extracted from building B2 were loaded at a constant velocity equalling 10 N/s. For mortar specimens collected from the remaining buildings, the velocity was 5 N/s. The loading of mortar specimens was carried out using testing machine Z100 (ZwickRoell AG, Ulm, Germany, accuracy class of testing machine 0.5; uncertainty of measurement 0.12%). The view of the specimen on the test stand is presented in Figure 6a. The results of the mortar strength tests are given in Table 2.

During the tests, the typical failure mode for the loading method for these types of specimens was observed. The cracks ran radially from the loaded part of the specimen (see Figure 6b). Due to the influence of confinement, the specimen fragments in the outline of steel punches took the form of a double cone after destruction (see Figure 6c). In total, 38 mortar specimens extracted from the bed joints of the brick walls were tested.

Before and after the DPT test, the maximum aggregate grains in the mortar specimens were measured. Such measurements were also conducted on the joint fragments left after cutting the specimens in the laboratory. The maximum measured dimension of the aggregate grains in the historical mortars was as follows: 12 mm—mortar in building B1, 10 mm—mortar in building B2, 6 mm—mortar in building B3 and 8 mm—mortar in building B4.

## 3. Discussion of Test Results

Table 3 presents the results of the penetrometer tests (PT), specifying the increases in the penetration depth corresponding to five impacts (*Δd_p (5)_*). The columns present the penetration depth for the impact ranges 1–5, 6–10, 11–15 and 16–20.

When analysing the results provided in Table 3, it may be stated that the highest penetration value is registered with the first five impacts, then regression and stabilisation follows.

The observed result is caused by the presence of a zone of degraded and weakened mortar material. The zone of degraded and weakened mortar is located at the surface of the masonry walls. For external walls, this is the consequence of the destructive impact of the external environment factors. For internal walls, the zone of degraded mortar arises during plaster removal. Furthermore, the conditions of the contact between the needle and the mortar surface at the beginning of testing, which are individual for each testing point, also exert some influence. 

Analysing the results of the conducted penetrometer tests, it may be stated that a zone of degraded mortar is observed in bed joints of all tested masonry buildings (see Table 3). This zone is further referred to in the article as the surface disturbance zone. In the surface disturbance zone, the penetration depth indications are distorted and should be omitted when the mortar strength is obtained. Therefore, the determination of a range of the surface disturbance zone is crucial in the analysis of the penetrometer test results. 

**Figure 7:**
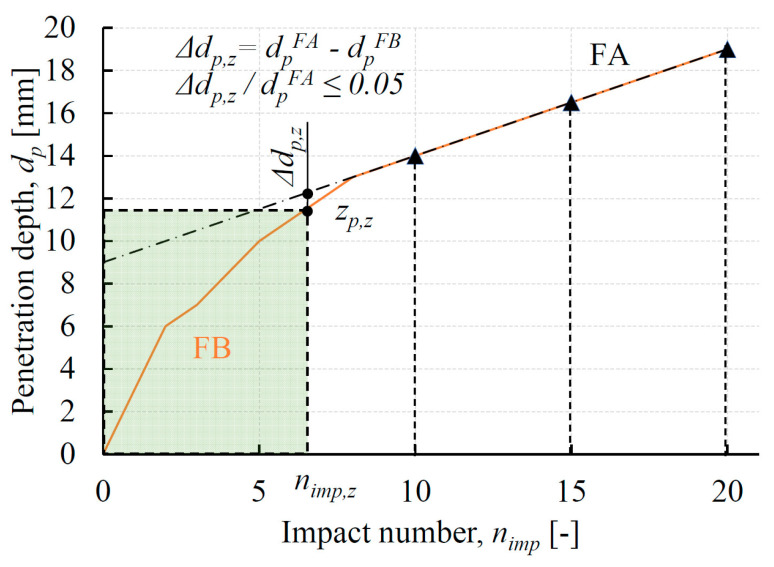
The manner of determining the range of the surface disturbance zone (*z_p,z_*) based on penetrometer test results. Where: FA—the linear approximation of the test results for *n_imp_* = 10, 15 and 20; FB—tests results.

The determination of the range of the surface disturbance zone (*z_p,z_*) should be conducted by means of the procedure presented in Figure 7. 

In the first step, the linear function FA is determined. The function FA is a linear approximation of the test results for *n_imp_* = 10, 15 and 20. The next step is to look for an *n_imp_* value (marked as *n_imp,z_*) for which the difference between the *d_p_*, determined on the basis of the FA function ($d_{p}^{FA}$) and directly from the tests ($d_{p}^{FB}$), is less than 5%. The value of penetration depth specified for *n_imp,z_* is the range of the surface disturbance zone (marked as *z_p,z_*).

The ranges of the surface disturbance zones (*z_p,z_*) in the bed joints of the tested historical walls, determined in line with the procedure provided in Figure 7, are presented in Table 4. The average values of the surface disturbance zones are from 9.1 to 14.5 mm. Table 4 also includes the number of impacts corresponding to the boundary of the surface disturbance zones (*n_imp,z_*). After rounding to integer values, the quantities of impacts *n_imp,z_* range from seven to eight.

**Figure 8:**
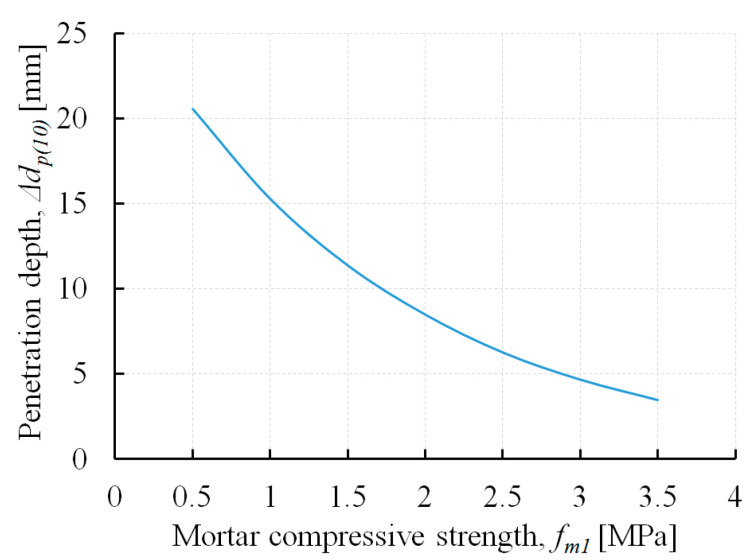
Relation between mortar compressive strength (*f*_*m*1_) and depth penetration after 10 impacts (*Δd_p (10)_*)—correlation curve according to [34].

In [34], a correlation curve was proposed for the evaluation of the mortar compressive strength based on the penetration depth of the penetrometer steel needle in the masonry bed joint. This correlation curve is presented in Figure 8. Mortar strength (*f*_*m*1_) can be determined considering the needle penetration depth after 10 impacts (Δ*d_p (10)_*). The greater the depth of penetration of the penetrometer steel needle after 10 impacts, the lower the mortar strength. Mortar strength, determined based on the conducted penetrometer tests and the mentioned correlation curve, is presented in Table 5.

Mortar compressive strength (*f*_*m*1_) was determined considering the measurement results registered outside the surface disturbance zones of bed joints. The compressive strength of historical mortars obtained using penetrometer tests were between 1.5 and 2.9 MPa.

The thickness of specimens in the DPT tests performed for four types of mortars was varied. The specimens cut out from the bed joints of building B1 were the thickest (average thickness 20.7 mm); whereas, the samples cut out from the joints of building B2 were the thinnest (average thickness 15.1 mm). The thickness of mortar specimens extracted from tested historical masonry were significantly higher than the thickness of mortar specimens cut out from contemporary masonry performed according to EN 1996-1-1 [37]. EN 1996-1-1 [37] recommends a thickness of bed joints in masonry from 6 to 15 mm. Based on this reasoning, the thickness of mortar specimens cut out from contemporary masonry ranged from a few millimetres at a minimum, to a maximum of 15 mm. 

**Figure 9:**
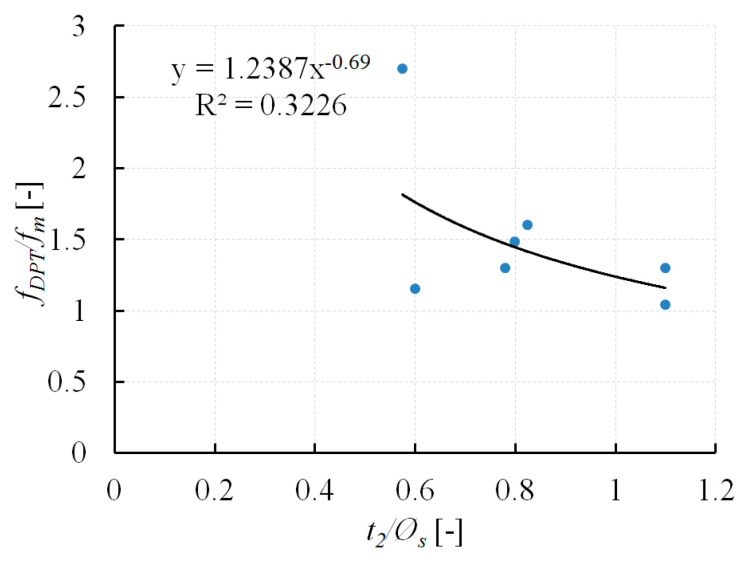
Relation between specimen slenderness (*t*_2_/*Ø_s_*) and value *f_DPT_*/*f_m_*.

The influence of the specimen thickness on the DPT test results and other related significant aspects were analysed in the studies [21,24,26,27,38]. As the specimens’ thickness increases, the impact of confinement caused by friction between the surface of the steel punch and the mortar specimens decreases. This affects the reduction in the ultimate compressive force of the specimen. The effect of reducing the ultimate compressive force, and thus the value *f_DPT_*, with the increase in specimen height, is presented in Figure 9.

The *f_DPT_* values refer to the compressive strength (*f_m_*) of the mortars obtained on standard mortar specimens with height and width values equalling 40 mm. The procedure for determining mortar compressive strength on standard specimens with a height and width of 40 mm is given in EN 1015-11 [14]. The results presented in Figure 9 consider the tests conducted on lime mortars and lime-cement mortars presented in [21,38]. The total sample thickness (*t*_2_), that is, the mortar sample thickness and the thickness of gypsum caps (see Figure 5), is considered. The conducted analysis provides a regression function in the following form:

**(*f_DPT_/f_m_*)* = *1.24(*t_*2*_/Ø_s_*)*^−*0.69*^* (1)

Based on the DPT test results, taking into account the sample height (*t*_2_), mortar compressive strength (*f*_*m*2_) may be calculated from the following relation:

*f_m*2*_ = f_DPT_/*(1.24(*t_*2*_/Ø_s_*)*^−*0.69*^*)** (2)

Table 6 presents mortar compressive strength values (*f*_*m*2_) determined from Equation (2).

It should be noted, however, that the coefficient of determination (R2) for the proposed function is low. Further research is needed for different mortar types to confirm the proposed relationships.

**Figure 10:**
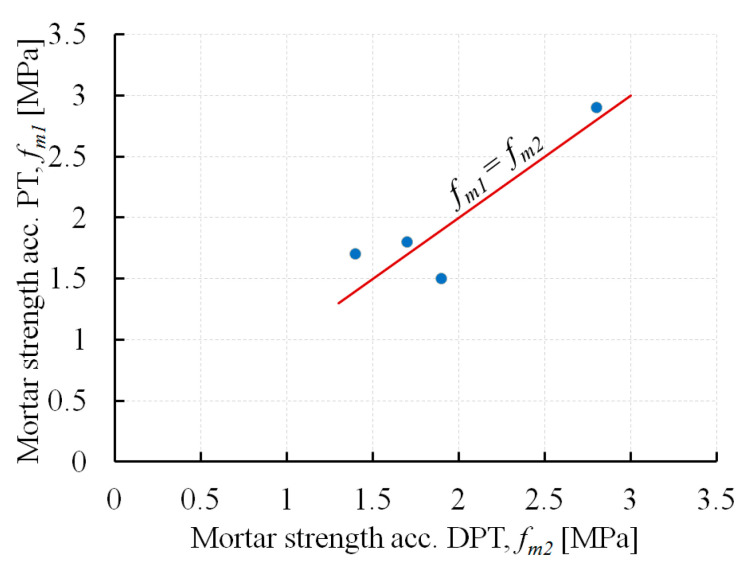
The comparison of the mortar compressive strength values determined based on penetrometer tests (*f*_*m*1_) and double punch tests (*f*_*m*2_).

Figure 10 compares the mortar compressive strength values based on penetrometer tests (*f*_*m*1_) and DPT tests (*f*_*m*2_).

The differences ranged from 4% to 27% (on average 14%). The mortar in the manufacturing hall building (B2) had the highest compressive strength. This was also confirmed in PT and DPT tests. In the remaining buildings, the mortars had compressive strength which ranged from 1.4 to 1.9 MPa.

## 4. Summary

This article presented the strength test results of the mortars used in the erection of four historical brick buildings. The mortars were tested in situ with the use of a penetrometer and in the laboratory by means of the DPT procedure. The laboratory tests were conducted on the specimens collected from the masonry bed joints. 

Tests showed that the historical mortars are characterised by specific properties, such as low strength, significant inhomogeneity and the presence of aggregate grains of large sizes considerably exceeding the current requirements. The characteristic properties of bed joints in the brick walls were their significant thickness and the presence of degraded material zones in the areas near the wall surface. The thickness of bed joints in tested masonry far exceeded the recommendations of current standards. Due to the above factors, modification of the applied testing methods and the methods of analysing their results was proposed in this paper. 

In regard to the penetrometer tests, the notion of a surface disturbance zone was introduced, and the manner of its range determination was provided.

The DPT test results were analysed, considering the influence of specimen thickness (see Equation (2)). The introduced modifications allowed for more precise determination of the compressive strength of mortar in the historical brick walls. 

The compressive strength of the tested historical mortars was minor. Based on the penetrometer tests, the mortar strength values ranged from 1.5 to 2.9 MPa, and the DPT tests ranged from 1.4 to 2.8 MPa.

The penetrometer method is simple and fast in application. Nevertheless, currently, there are not a sufficient number of penetrometer tests carried out on historical buildings in order to verify this method for different types of mortars.

The DPT method requires cutting out samples from wall bed joints and conducting laboratory tests, which is more complicated. Cutting out a significant quantity of samples is sometimes impossible for conservation reasons. 

For the reasons given above, the authors of this paper suggest the compilation of both testing methods in order to establish the mortar compressive strength in the bed joints of historical buildings. The penetrometer tests allow limiting the sampling of structures. The results of the DPT should be used for the verification of the mortar strength specified in the penetrometer tests.

## Figures and Tables

**Table 1:** Characteristics of buildings.

Building Symbol	Building Function	Number of Stories	Construction Year	Wall Material
B1	warehouse	1	1882	brick masonry
B2	manufacturing hall	1	1907	brick masonry
B3	tenement house	2 + 1 ^1^	1909	brick masonry
B4	tenement house	2 + 1 ^1^	1910	brick masonry

^1^ building with basement.

**Table 2:** Results of double punch tests (DPT) on mortar specimens extracted from bed joints of historical masonry.

Building	Number of Specimens (pcs.)	Average Mortar Specimen Thickness ^1^ (mm)	*f_DPT_*			
			Value Range (MPa)	Average Value (MPa)	Standard Deviation (MPa)	Coefficient of Variation (–)
B1	8	20.7 (25.7)	1.02–2.80	1.98	0.52	0.26
B2	9	15.1 (19.0)	3.10–4.46	3.59	0.47	0.13
B3	9	17.1 (23.2)	0.92–2.06	1.48	0.33	0.22
B4	12	16.8 (20.5)	1.33–2.69	2.00	0.44	0.22

^1^ The specimen total thickness is shown in brackets (with the gypsum caps)—marked as *t*_2_ in Figure 5.

**Table 3:** The list of the penetrometer test results.

Building	*Δd_p (5)_* (mm)			
	1–5	6–10	11–15	16–20
B1	(7.0–12.0) 9.0	(5.0–7.0) 6.3	(5.0–9.0) 6.3	(5.0–7.0) 6.0
B2	(5.5–10.5) 8.0	(1.5–4.0) 2.7	(1.0–3.0) 2.2	(1.5–2.5) 2.1
B3	(6.0–14.5) 10.5	(4.0–7.0) 6.1	(3.0–7.0) 5.0	(3.0–5.0) 4.1
B4	(6.0–12.0) 9.4	(3.5–8.0) 5.1	(2.5–9.0) 5.1	(2.5–8.0) 4.4

In brackets—the range values. Outside the brackets—the average value.

**Table 4:** The ranges of the surface disturbance zones (*z_p,z_*) and the quantities of impacts corresponding to the zone boundary (*n_imp,z_*).

Building	*z_p,z_* (mm)		*n_imp,z_* (mm)	
	Value Range	Average Value	Value Range	Average Value ^1^
B1	5–16	11.2	2–10	7.0 (7)
B2	6–11	9.1	5–9	6.5 (7)
B3	10–18	14.5	7–9	7.8 (8)
B4	8–15	11.4	4–10	6.7 (7)

^1^ The brackets include *n_imp,z_* after rounding to integer values.

**Table 5:** The mortar compressive strength (*f*_*m*1_) determined based on the penetrometer test results according to the correlation curve from [34].

Building	*f*_*m*1_ (MPa)	
	Value Range	Average Value
B1	1.0–1.9	1.5
B2	2.5–3.7	2.9
B3	1.2–2.3	1.7
B4	0.9–2.8	1.9

**Table 6:** Mortar compressive strength values (*f*_*m*2_) determined based on the DPT test with consideration of Equation (2).

Building	*t_*2*_/Ø_s_* (–)	*f*_*m*2_ (MPa)
B1	1.29	1.9
B2	0.95	2.8
B3	1.16	1.4
B4	1.03	1.7
